# Peridotite weathering is the missing ingredient of Earth’s continental crust composition

**DOI:** 10.1038/s41467-018-03039-9

**Published:** 2018-02-12

**Authors:** Andreas Beinlich, Håkon Austrheim, Vasileios Mavromatis, Ben Grguric, Christine V. Putnis, Andrew Putnis

**Affiliations:** 1 The Institute for Geoscience Research (TIGeR), Department of Applied Geology, Curtin University, Perth, WA 6845 Australia; 2 Physics of Geological Processes (PGP), University of Oslo, 0316 Oslo, Norway; 3 Géosciences Environnement Toulouse (GET), CNRS UMR5563, Observatoire Midi–Pyrénées, 14 Av. E. Belin, 31400 Toulouse, France; 4 Centre for Exploration Targeting (CET), The University of Western Australia, 35 Stirling Highway, Perth, WA 6009 Australia; 5 Institut für Mineralogie, Universität Münster, 48149 Münster, Germany; 6 Department of Chemistry, Curtin University, Perth, Australia

## Abstract

The chemical composition of the continental crust cannot be adequately explained by current models for its formation, because it is too rich in Ni and Cr compared to that which can be generated by any of the proposed mechanisms. Estimates of the crust composition are derived from average sediment, while crustal growth is ascribed to amalgamation of differentiated magmatic rocks at continental margins. Here we show that chemical weathering of Ni- and Cr-rich, undifferentiated ultramafic rock equivalent to ~1.3 wt% of today’s continental crust compensates for low Ni and Cr in formation models of the continental crust. Ultramafic rock weathering produces a residual that is enriched in Ni and also silica. In the light of potentially large volumes of ultramafic rock and high atmospheric CO_2_ concentrations during the Archean, chemical weathering must therefore have played a major role in forming compositionally evolved components of the early Earth’s crust.

## Abstract (web summary)

The concentration of Ni and Cr of the continental crust cannot be explained by formation models involving differentiated magmatic rocks. Here, the authors show that hydrothermal alteration and chemical weathering of ultramafic rock compensates for the low Ni and Cr concentrations of island arc-type magmatic rocks.

## Introduction

**Fig. 1:**
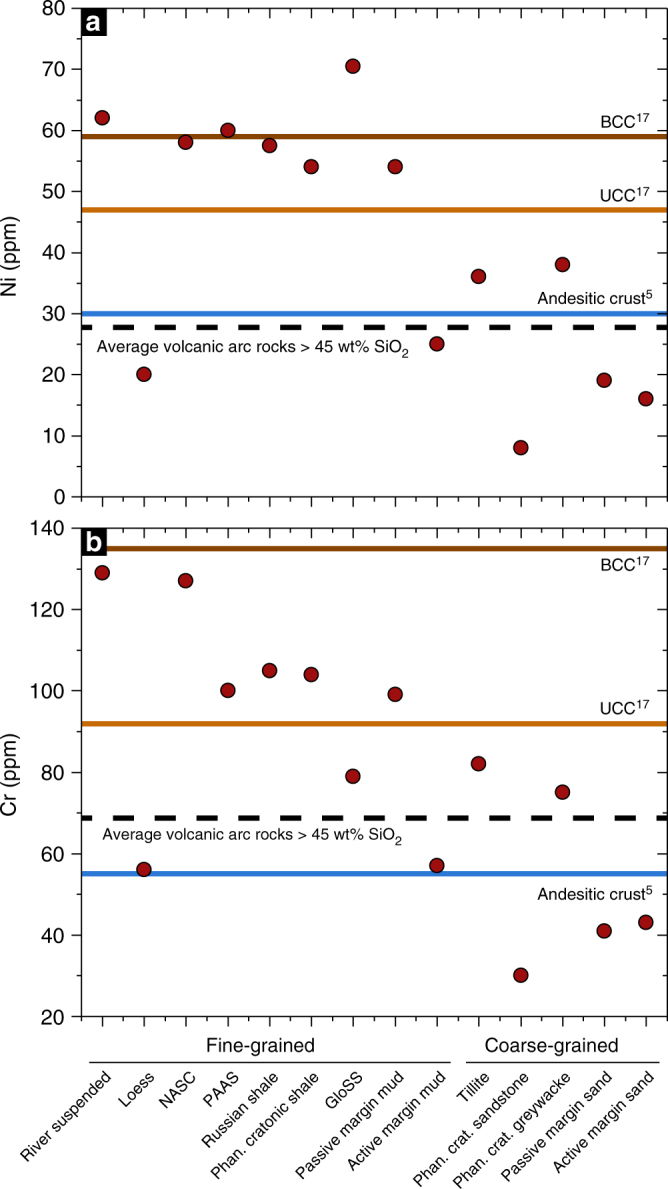
Ni and Cr concentrations in continental sediments. Ni (**a**) and Cr (**b**) compositional data for various fine-grained and coarse-grained continental sediments based on the compilation in McLennan^21^ relative to the Ni and Cr concentrations of the average andesitic crust^5^, upper (UCC) and bulk (BCC) continental crust^17^, and average volcanic arc (dashed black lines). NASC = North American shale composite, PAAS = Post-Archean average Australian shale, GloSS = average global subducting sediment

The Earth’s continental crust is a unique chemical environment due to the presence of liquid water and its highly evolved chemical composition. It also represents the most accessible part of our planet, yet its formation mechanism remains enigmatic^1^. Models of continental crust formation involve partial melting of mantle peridotite^2^ and have developed from amalgamation of island arc-type magmatic rocks of andesite composition^3–5^, magmatic recycling of chemically weathered material^6^, to geodynamically more complex mechanisms involving melting of mantle metasomatites^7,8^, reactions with overlying peridotite^9,10^ and differentiation of subducted material via buoyancy-driven delamination–relamination processes^11,12^. Model complexity has progressively increased to account for the mismatch between experimentally derived data, the composition of juvenile island-arc type andesitic melts and the composition of the continental crust^13^. Despite the general consensus among the different estimates of the continental crust composition, the concentrations of Ni (bulk continental crust: 59 ppm; upper continental crust: 47 ppm) and Cr (bulk continental crust: 135 ppm; upper continental crust: 92 ppm) do not reconcile with the average andesitic composition of 30 ppm Ni and 55 ppm Cr^1,5,13–19^ (Fig. 1). Consequently, crustal formation models lack a component that balances Ni and Cr concentrations although these models are otherwise consistent with the composition of average andesite. High concentrations of Ni and Cr are almost exclusively found in undifferentiated ultramafic rock, typically up to 4000 ppm^20^, indicating that transfer of Ni and Cr to the continental crust is incompatible with partial melting of peridotite. In contrast, elevated Ni and Cr concentrations in continental sediments^21^ relative to the average andesitic and upper continental crust indicate their addition by weathering and erosion processes (Fig. 1). Here we demonstrate how ultramafic material becomes part of the continental crust via hydrothermal carbonation and subsequent chemical weathering and erosion by analysing hydrothermally altered, weathered and redeposited ultramafic material from Western Australia and Norway. If the surface processes operate on sufficiently long time scales, the compositional heterogeneity, introduced by tectonic emplacement of isolated ultramafic occurrences within the otherwise felsic continental crust, will be effectively homogenised. Furthermore, fluid-driven ultramafic rock alteration and weathering favour the production of Si-enriched product phases extending to pure quartz^22–25^. Distinct Ni concentrations of the upper continental crust, volcanic arcs and ultramafic rocks provide constraints on the contribution of ultramafic rock alteration to continental crust composition. Due to the undeniable coexistence of aqueous fluid and ultramafic rock at the interface between lithosphere, hydrosphere and atmosphere possibly since the Hadean^26^ and particularly during the Archean, we propose a re-evaluation of the role of ultramafic rock alteration to the compositional evolution of the continental crust.

## Results

### Ultramafic rock alteration

The presence of ultramafic rock in the Earth’s crust is intrinsically linked to its unique geodynamic evolution and forms a compositionally distinct component characterised by high Mg, Ni and Cr and low Si concentrations. Ultramafic rocks occur in the continental crust in ophiolite complexes, komatiite magma flows and ultramafic intrusions of dominantly Archaean age, mantle xenoliths in basalt and as the result of hyperextended passive continental margins^27^. As a major component of the Earth’s mantle, ultramafic rock equilibrates under dry, high-temperature conditions and is thus metastable at the Earth’s surface and in the presence of aqueous fluid. Alteration of ultramafic rock proceeds by dissolution of primary olivine and pyroxene and precipitation of secondary phases in response to the alteration temperature and pressure conditions and fluid composition. Mineral replacement reactions, driven by hydrothermal fluid and weathering processes, significantly affect rock-physical properties, have been linked to the formation of life-essential building blocks and represent an important sink in the global carbon cycle^28–31^ but have not been considered important for continental crust formation.

**Fig. 2:**
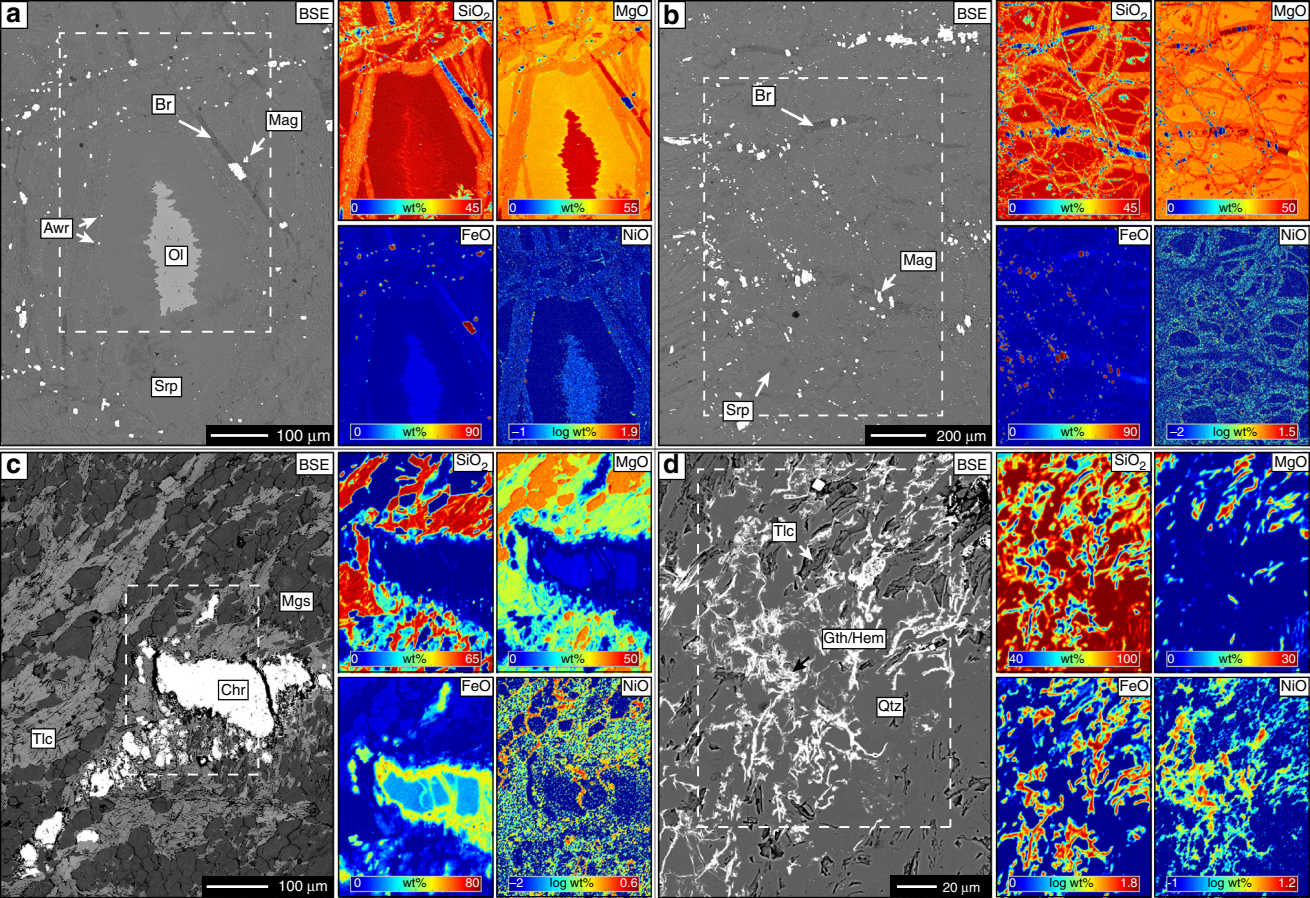
Textural and chemical changes during ultramafic rock alteration. Back-scattered electron (BSE) images and element distribution maps of **a** serpentinised peridotite, **b** serpentinite, **c** talc-carbonate altered serpentinite, and **d** laterite show that the reaction sequence is accompanied by progressive partitioning of Si and Ni into weathering resistant phases. **a**, **b** show samples from the Feragen Ultramafic Body (Norway), **c**, **d** show samples from the Six Mile Well-Goliath Complex, SGC (Australia). For the discussion of different alteration environments we distinguish between weathering that happens exclusively at the surface (e.g. laterite formation) and hydrothermal alteration (e.g. serpentinisation, carbonation) at depth and at higher temperature. Awr = awaruite, Br = brucite, Chr = chromite, Gth = goethite, Hem = hematite, Mag = magnetite, Mgs = magnesite, Ol = olivine, Qtz = quartz, Srp = serpentine, Tlc = talc

Serpentinisation is an alteration process in ultramafic rock, driven by dominantly aqueous fluid, commonly in the course of seawater circulation through the oceanic lithosphere and results in replacement of primary olivine ((Mg,Fe)_2_SiO_4_) by secondary serpentine phases (Mg_3_Si_2_O_5_(OH)_4_) and magnetite (Fe_3_O_4_) (Fig. 2a, b). Concomitant precipitation of brucite (Mg(OH)_2_) and awaruite (Ni–Fe alloy) as reaction by-product phases is controlled by fluid pH, *f*_O2,aq_ and *a*_SiO2,aq_, that are dependent on temperature and rock composition. Hydrothermal ultramafic rock alteration by CO_2_-enriched fluid in the continental crust (e.g. during ophiolite alteration) promotes precipitation of additional magnesite (MgCO_3_) and dolomite (CaMg(CO_3_)_2_). Carbonate precipitation increases *a*_SiO2,aq_ and commonly stabilises additional secondary silicate minerals with Si/(Mg+Fe) higher than in the precursor olivine and/or serpentine (Fig. 2c). Along an idealised isothermal reaction path of increasing *a*_CO2,aq_ peridotite will alter to serpentinite, followed by the ophimagnesite assemblage (serpentine+magnesite/dolomite), soapstone (talc+magnesite/dolomite) and listvenite (quartz+magnesite/dolomite)^22,23,25^. Isobaric cooling at constant *a*_CO2,aq_ will result in a similar reaction path with additional clay mineral formation at low temperature and high *a*_CO2,aq_ (Supplementary Fig. 1).

**Fig. 3:**
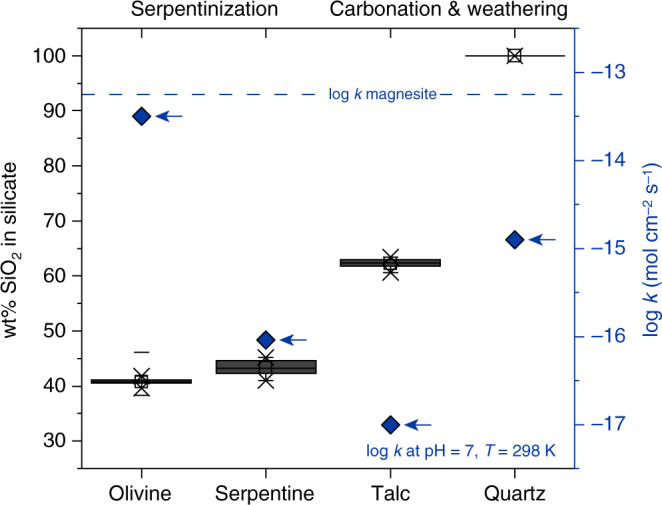
Solubility of minerals involved in CO_2_ alteration of ultramafic rock. SiO_2_ content and dissolution kinetics of the major silicate phases occurring during ultramafic rock alteration compared to the dissolution rate of magnesite at pH 7 and 25 °C. The dissolution rate (diamond markers) is indicated on the right-hand-side axis. The size of the boxes reflects the interquartile range of mineral compositions analysed in this study. Dissolution rates: olivine^75^, serpentine^76^, talc^77^, quartz^78^, magnesite^79^

**Fig. 4:**
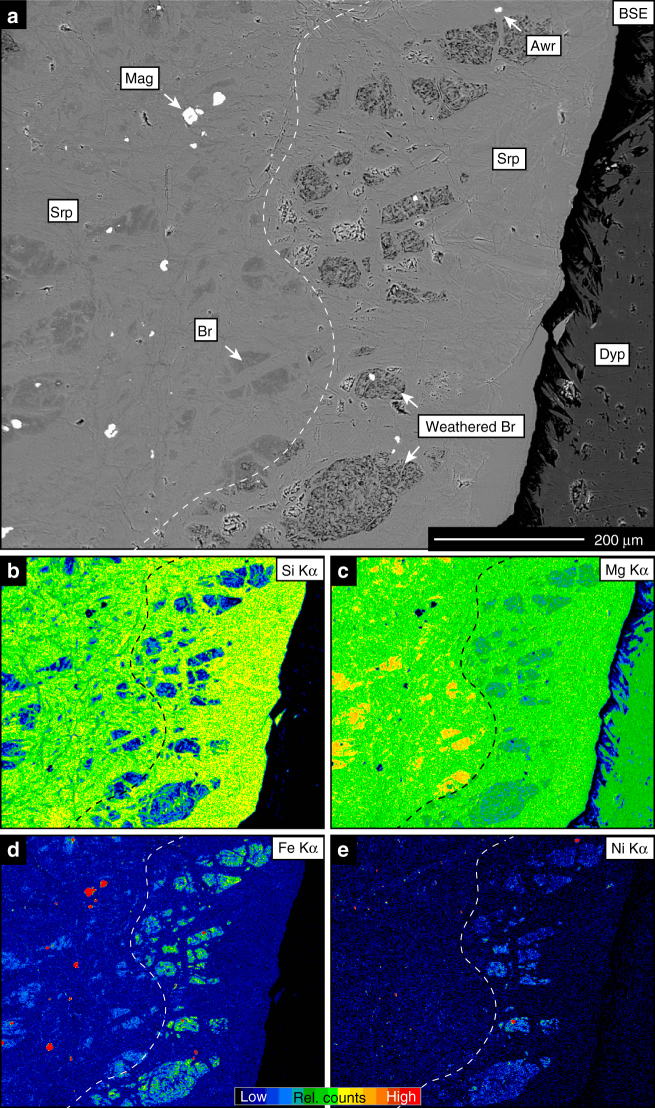
Element distribution in low-temperature altered peridotite. Back-scattered electron (BSE) image (**a**) and qualitative element maps (**b**–**e**) across a cold-temperature weathering front in serpentinised peridotite from Feragen, eastern Norway. Weathering results in preferential dissolution of brucite from the weathered part of the sample (right hand side of the weathering front). Sites previously filled with brucite are porous and enriched in Ni and Fe. The dashed line indicates the approximate position of the weathering front. Secondary Mg-carbonate (dypingite) has formed on the surface. Br = brucite, weathered Br = site of brucite dissolved during weathering, Dyp = dypingite surface efflorescence, Mag = magnetite, Srp = serpentine

**Fig. 5:**
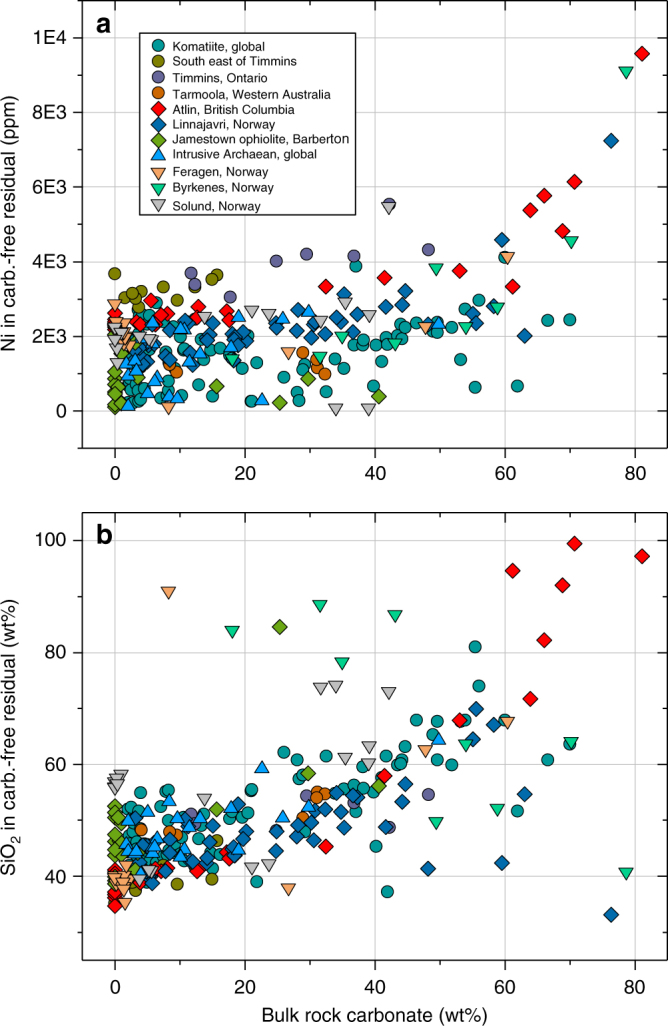
Ni and SiO_2_ enrichment trend during weathering of hydrothermally altered ultramafic rock. **a** Ni and **b** SiO_2_ concentrations in carbonated ultramafic rock samples are recalculated on a CO_2_-free basis to reveal the effect of kinetically favoured breakdown of carbonate during weathering. The data set includes Archaean komatiite and intrusive peridotite samples (circles and upward triangle), alpine peridotite (diamonds), and sediment–hosted peridotite boulders from Norway (downward triangles). Recalculation of data was done by subtracting bulk rock CO_2_ and renormalisation to 100 wt%. Data are compiled from refs. ^22,25,47,57–59,80^, unpublished data from Feragen, Solund, Byrkenes and Linnajavri are listed in Supplementary Data 1

Surface weathering of fresh and altered peridotite proceeds in response to climatic conditions, fluid composition and reaction kinetics of its constituents. Weathering of serpentinised and carbonated peridotite causes preferential breakdown of brucite and carbonate, respectively, while relicts of serpentine and talc are often preserved at deeper levels of the weathering profile and in colder climates (Figs. 2d, 3 and 4). For example, dissolution of magnesite at surface weathering conditions is approximately 3.75 orders of magnitude faster than dissolution of talc (Fig. 3). Common secondary phases include hydrous Mg-carbonate, clay minerals, oxide and hydroxide-compounds of ferric iron, and silica phases. Additional porosity generation in weathered rock surfaces is the result of open-system net-dissolution without precipitation of secondary phases and of mechanical removal of weathering products. Replacement reactions during cold climate weathering are typically restricted to the outermost parts of the rock, as exemplified by currently weathering peridotite from eastern Norway^30,32^, and produce sharp reaction interfaces with the fresh interior (Fig. 4). In contrast, tropical weathering results in the formation of deep laterite horizons as e.g. in New Caledonia and Western Australia^33,34^. Nevertheless, in both cases the weathering residual is characterised by high concentrations of Ni, Si, Fe and depletion in Mg (Fig. 4). Prolonged tropical weathering often results in the formation of silica-rich hard pans near the surface, resembling jasper, a rock composed predominantly of silica with some hematite^24^ (Fig. 2d), and economically significant Ni laterite deposits overlying the ultramafic bedrock. Figure 5 shows the compositional change of hydrothermally carbonated peridotite from komatiite sequences, ophiolites and sedimentary basins in response to carbonate breakdown during chemical weathering. Carbonate removal by surface weathering increases both Ni and SiO_2_ to values that are consistent with deposit grades and the composition of continental crust, respectively (Supplementary Data 1).

### Natural alteration sequences

Ultramafic rock alteration sequences involving carbonation and weathering are common on all continents and allow for assessment of compositional changes along the alteration path. We analysed samples from the Agnew-Wiluna greenstone belt in the Archean Yilgarn craton of Western Australia, and serpentinised, weathered and eroded alpine peridotite from southern Norway.

The Six Mile Well-Goliath Complex (SGC), near Yakabindie Station from the Western Australian Yilgarn Craton^35^, provides unique insight into the effects of tropical weathering of previously talc-carbonate altered serpentinite and the links between bulk rock geochemical changes and distinct steps along the alteration path. The SGC is one of numerous type-2 Ni deposits hosted in intensely carbonated serpentinite. The alteration precursor has been interpreted as a giant, sub-seafloor olivine cumulate intrusion into intermediate to felsic volcanic rocks^35^. The 120 m deep drill core studied here intersects an upper 25 m thick horizon of laterite, a 75 m thick zone of massive talc-carbonate and carbonate-altered serpentinite above pristine serpentinite at 100 m depth. One felsic and one mafic dike intersect the investigated drill core at 78 and 45 m depth, respectively. In thin section, serpentinite samples display a typical mesh texture, after primary olivine, composed of serpentine veins and brucite-serpentine intergrowths in mesh centres. Additional Ni-sulphide (millerite, heazlewoodite, godlevskite) and chromite are present as minor matrix constituents. Magnesite veins and patches increase in abundance towards the talc-carbonate alteration zone in the shallower part of the drill core. Talc-carbonate alteration of serpentinite is massive and resulted in the formation of coarse-grained magnesite in a matrix composed of talc, minor chlorite and magnetite, consistent with the assemblages of soapstone altered peridotite elsewhere (Fig. 2c)^25^. Cr-spinel from the precursor serpentinite is altered to ferrichromite. The influence of near surface weathering becomes apparent at ~25 m depth as a gradual increase in the abundance of ferric iron compounds and quartz. The uppermost 25 m of the core are composed of fine-grained, red-stained intergrowths of hematite, goethite and quartz. Talc relicts are commonly preserved (Fig. 2d), whereas magnesite has not been found in the laterite.

**Fig. 6:**
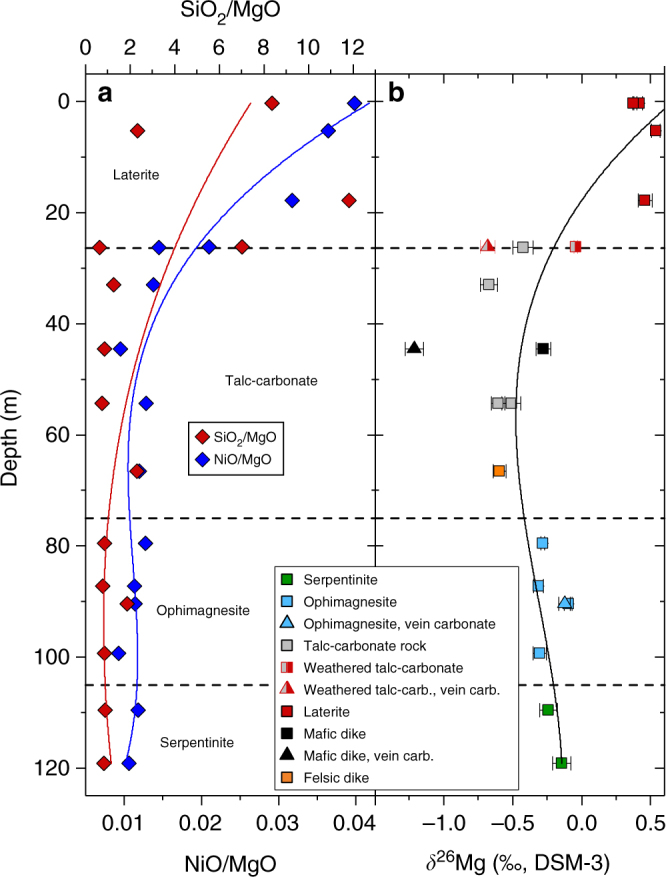
Bulk rock chemical changes in drill core through weathered and hydrothermally altered peridotite. Distinct changes in **a** NiO/MgO and SiO_2_/MgO and **b** *δ*^26^Mg in laterite and talc-carbonate altered serpentinite from the SGC are indicative of open system alteration. Mg depletion in the laterite accompanied by increasing *δ*^26^Mg is related to preferential breakdown of magnesite during weathering, whereas deviation of *δ*^26^Mg from the background value (serpentinite) in deeper parts of the profile reflects addition of ^24^Mg during hydrothermal carbonation. Error bars reflect the 2*σ* standard deviation. Data are listed in Supplementary Tables 2 and 3

The composition of serpentinite samples from the SGC is consistent with that of abyssal peridotite and characterised by low rare earth element (REE; <8 ppm) and high Ni and Cr (1200–3800 ppm) concentrations and a slightly elevated MgO/SiO_2_ (1.18 ± 0.04)^20^ (Supplementary Data 2). Talc-carbonate altered samples show a major and trace-element signature that is relatively similar to the precursor serpentinite except for enrichment in Ce and depletion in S. In contrast, the overlying laterite is depleted in Mg resulting in distinctly elevated SiO_2_/MgO and NiO/MgO (Fig. 6a). Elevated concentrations of Y, Ba and La at the surface indicate sediment input from nearby felsic bedrock. Formation of the 25 m thick, near-surface laterite is ascribed to weathering of talc-carbonate altered serpentinite in a warm and humid climate prevailing in Western Australia between the mid-Mesozoic and early-middle Tertiary^33^.

**Fig. 7:**
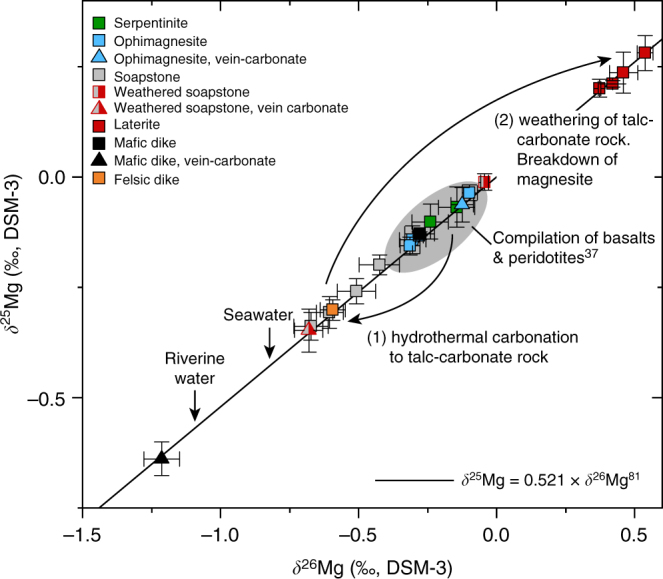
Mg isotope systematics in ultramafic alteration and weathering systems. Three-isotope plot showing Mg isotope ratios of bulk rock samples from the SGC drill core through weathered (laterite) and hydrothermally carbonated (talc-carbonate) serpentinite. Mg isotope ratios of the precursor serpentinite are consistent with the compilation of pristine ultramafic and mafic rocks^37^. Talc-carbonate altered rocks are enriched in ^24^Mg, which is interpreted to reflect addition of ^24^Mg during open system hydrothermal alteration. Preferential breakdown of magnesite in laterite weathered parts of the drill core results in an overall depletion in bulk rock Mg and effective removal of ^24^Mg. The laterite Mg isotope ratios are controlled by high *δ*^25,26^Mg values of relict talc and are consistent with the composition of talc elsewhere^36^. *δ*^25^Mg and *δ*^26^Mg values are consistent with mass-dependent Mg isotope fractionation [81]. Error bars reflect the 2*σ* standard deviation. Data are listed in Supplementary Table 1

Interpretation of bulk rock compositional changes due to pervasive open-system hydrothermal alteration is often complicated by significant volume changes, the lack of a robust reference frame for mass-balance calculations and potential compositional heterogeneity of the precursor rock. However, the stable isotope ratio of Mg (*δ*^26^Mg) is independent of earlier magmatic processes and Mg fractionates into ^24^Mg-enriched magnesite and ^26^Mg-enriched talc during hydrothermal peridotite carbonation^36,37^. Thus, bulk rock concentrations of Mg in combination with its isotope ratios provide constraints on elemental mass-changes related to stabilisation and breakdown of individual mineral phases. Deviation of the Mg isotope ratios of talc-carbonate altered samples to values lower than in the precursor serpentinite at near constant SiO_2_/MgO is consistent with addition of small amounts of ^24^Mg during serpentinite carbonation (Figs. 6b and 7; Supplementary Table 1). In contrast, the distinct increase in laterite *δ*^26^Mg, SiO_2_/MgO and NiO/MgO indicates preferential breakdown of earlier formed hydrothermal magnesite thus forming an Si- and Ni-rich weathering residual with Mg isotope ratios controlled by weathering-resistant talc (Fig. 3). Textural analysis of the laterite samples reveals abundant pore space after magnesite and corroborates the presence of talc relicts in a quartz matrix together with a fine-grained network of hematite and goethite (Fig. 2d). Hydrothermal carbonation had strong influence on the mineralogy and chemical composition of the weathering residual by partitioning Si and Ni into slowly dissolving sheet silicate, the latter also in Fe-compounds and most of the Mg in soluble carbonate (Fig. 3). Thus high Ni concentrations in laterite form without addition of externally derived Ni and may also involve Ni mobilisation resulting from dissolution of Ni-bearing sheet silicate and carbonate phases during weathering. Weathering of pristine peridotite predominantly involves breakdown of mainly olivine and can be expected to result in relatively congruent element release as opposed to weathering of hydrothermally carbonated peridotite.

Estimates of the crustal composition are based on large sets of sediment samples that integrate over the composition of large provenance areas^4,5,17^. Hence, evaluating the contribution of ultramafic rock alteration to the continental crust composition requires examination of sediments that were sourced from catchment areas containing peridotite. Relatively fast dissolution kinetics and mechanical disintegration rates of ultramafic material during erosion and transport result in only few continental sedimentary basins with documented ultramafic detritus^24,38^. The Devonian continental sedimentary basins of southern Norway (Feragen, Solund, Byrkenes) thus provide unique natural laboratories to investigate the effect of erosion of weathered peridotite on bulk sediment composition.

Samples used to investigate the influence of peridotite in the sediment provenance area on bulk sediment composition were collected from the Solund Devonian sedimentary basin and from a Devonian conglomerate-dominated sedimentary sequence in the periphery of the Feragen ultramafic body^24,32^. The Solund basin is one of five continental supra-detachment fault basins situated along the coast of south-western Norway. Caledonian nappes containing the Solund-Stavfjord ophiolite constitute the depositional basement of the Solund basin and are separated from the underlying Western Gneiss region by the Nordfjord-Sogn detachment zone. The Lower Devonian depositional age of the Solund basin has been constrained by fish and plant fossils, at which time Scandinavia was in a near-equatorial position, promoting tropical weathering of the exposed ophiolite^39,40^. The basin infill is dominated by conglomerate with subordinate sandstone layers that are distinctly more abundant in the central part of the basin. The conglomerate layers contain between 1 and 20 vol% peridotite boulders that show an alteration-related zonation with peridotite cores, intermediate talc zones and red rims composed of a quartz+hematite ± calcite assemblage^24^ that are similar to those observed for weathering of talc-carbonate rock at the SGC, Western Australia. Alteration involved a strong depletion in bulk rock Mg and formation of a quartz-hematite-dominated weathering assemblage resembling jasper in the outer layer, while the cores often retain the original peridotite. Some former peridotite boulders have been entirely silicified and/or carbonated and can only be classified as former peridotite based on their high Cr and Ni contents.

**Fig. 8:**
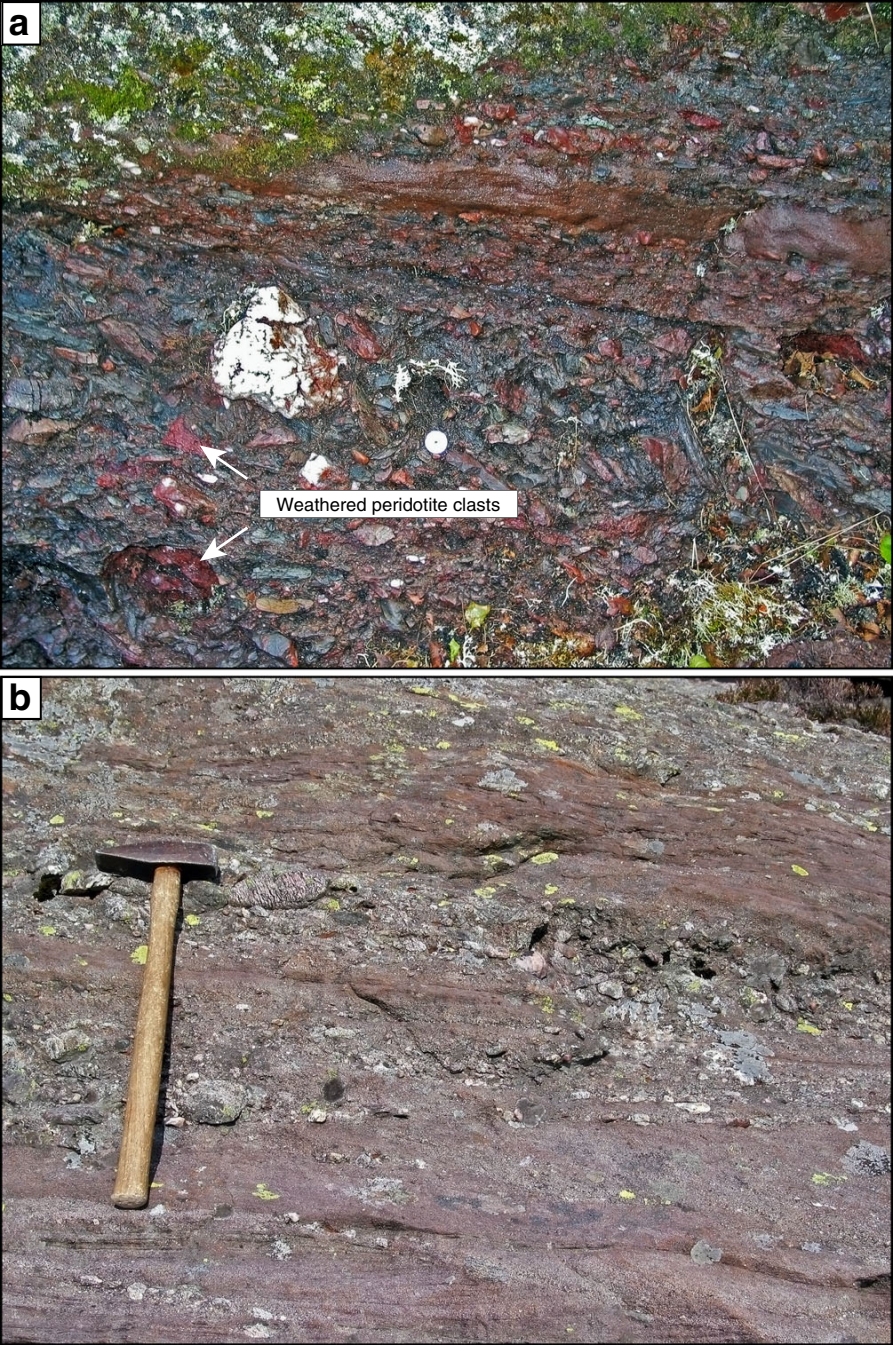
Outcrop photographs of ultramafic detritus-bearing Devonian sediments. **a** Coarse-grained conglomerate in the Feragen basin containing abundant weathered serpentinized peridotite. Red clasts resembling jasper are weathered peridotite and often contain serpentinite and peridotite in the core. **b** Sandstone layer in the Solund basin. Sandstone layers from both locations are strongly enriched in Ni and Cr but otherwise consistent with the composition of the continental and andesitic crust

Sandstone layers interbedded with conglomerate are abundant, display a distinctive red colour and comprise a mixture of ultramafic, mafic and felsic provenance (Fig. 8). Mafic clasts are often altered to epidote and occur together with boulders composed of peridotite-derived quartz-hematite intergrowths within the sediment matrix mainly composed of quartz and plagioclase with minor almandine garnet. Almandine is sometimes replaced by talc in the vicinity of larger weathered peridotite boulders and individual chromite grains are frequently replaced by chlorite and magnetite or hematite.

**Fig. 9:**
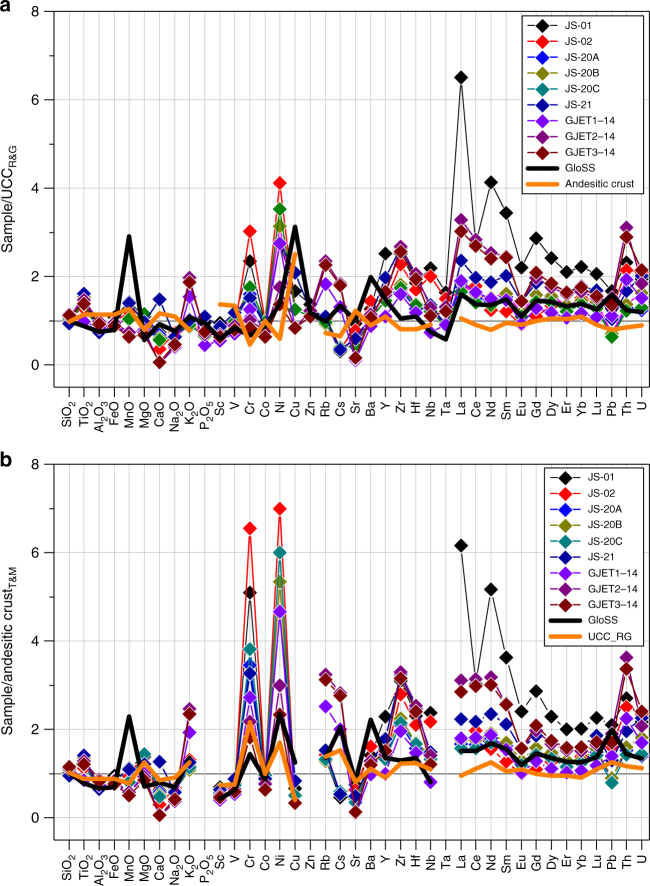
Normalisation plots of Norwegian peridotite-bearing sandstone samples. Compositions are normalised to **a** the upper continental crust (UCC)^17^ and **b** average andesitic crust^5^. The composition of the global subducting sediment (GloSS)^41^ is shown for comparison. The distinct enrichment in Ni and Cr of sediment samples is complementary to their relatively low concentrations in andesite. The different Cr/Ni in UCC and GloSS in **b** may suggest preferential Ni particulate transport into the oceans^46^. The composition of sediment samples is listed in Supplementary Table 2

The composition of sandstone samples from the Solund and Feragen sedimentary basins (Fig. 8) aligns with that of the bulk continental crust^17^ except that it is distinctly enriched in Ni, Cr and light REE (LREE) (Supplementary Table 2). For example, the average SiO_2_ content is 63 ± 3.76 wt% and the molar Mg# (Mg/Mg+Fe) is 0.35. Chondrite-normalised REE patterns are steep from La to Gd, flat for the heavy REE and show a slight negative Eu anomaly (Supplementary Fig. 2). The relatively high LREE concentrations are related to the presence of abundant LREE-rich epidote replacing mafic detritus. The effect of ultramafic input into sedimentary basins can be highlighted by normalising the composition of peridotite-bearing sandstone samples to the composition of the upper continental and average andesitic crust (Fig. 9)^5,17^. Relative to the upper continental crust, sandstone samples from Norway show a distinct enrichment in Ni and Cr that matches their depletion in the average andesitic crust (Fig. 9a). The enrichment becomes more apparent, when both the upper continental crust and Norwegian sandstone samples are normalised to the average andesitic crust^5^ (Fig. 9b). Fig 9 also shows the composition of the global subducting sediment (GloSS)^41^, that represents the terrigenous sediment runoff into the region of melt production beneath volcanic arcs and broadly resembles the composition of the upper continental crust. Andesite-normalised compositions of GloSS and bulk continental crust imply that Ni-bearing detritus is preferentially removed from the continental crust over Cr.

Excellent exposure and clear field relationships of peridotite, serpentinite and their weathering products at the Feragen Ultramafic Body (FUB), south eastern Norway, allow for detailed sampling and textural analysis of mineral replacement interfaces, whereas in the SGC this is complicated by the large scale of the drill core and lack of spatial context. The circular FUB (~4.1 km in diameter) is one of several ultramafic intrusions in the Cambro-Silurian felsic basement of the Trondheim basin and composed of interlayered dunite and harzburgite^30,32,42^. Its central part is relatively unaltered and the degree of serpentinisation increases towards the contact with the surrounding country rocks. Rock surfaces exposed to weathering display sharp, alteration-related discoloration fronts typically reaching ~2 cm into the fresh rock. In contrast to the SGC drill core, primary olivine is ubiquitously preserved and serpentine veins are often intergrown with brucite. Abundant µm-sized awaruite grains rim the contact between mesh cells and serpentine veins and are almost absent from mesh centres in the vicinity to olivine−serpentine interfaces (Fig. 2a, b). Weathering fronts are defined by the breakdown of brucite, which creates porosity and nucleation sites for secondary pyroaurite and hydrous Mg-carbonate phases (dypingite, lansfordite) in the outer rock layers (Fig. 4).

## Discussion

Primary olivine contains on average ~2880 ± 410 ppm Ni and represents the dominant Ni host mineral in peridotite^43^. Analysis of serpentinisation textures reveals that Ni in olivine partitions into secondary Fe-Ni alloy (awaruite) or Ni-sulphide phases (e.g. heazlewoodite), depending on the sulphur fugacity during serpentinisation (Fig. 2a). Additionally, low Ni concentrations are measurable in serpentine and magnetite. The common presence of awaruite in serpentinite attests to the reducing geochemical conditions during serpentinisation. Micrometre-sized awaruite grains are typically aligned along the original grain boundary of the olivine. Serpentine veins, representing initial pathways for the alteration fluid, often show elevated Ni concentrations relative to serpentine in the mesh centres (Fig. 2a, b). Talc-carbonate altered serpentinite samples from the SGC are devoid of awaruite, which has generally not been documented from talc-carbonate altered ultramafic rock elsewhere suggesting that *f*_O2,aq_ and/or *a*_SiO2,aq_ during carbonation of peridotite and serpentinite are unfavourable for awaruite formation and preservation. Nickel released from breakdown of awaruite and serpentine due to hydrothermal carbonation primarily partitions into talc reaching concentrations similar to that in primary olivine^43,44^, while Ni concentrations in magnesite are below the detection limit (~0.03 wt%) (Fig. 2c, Supplementary Table 3). Fast dissolution rates and high *f*_O2,aq_ during near-surface weathering in humid climate zones cause preferential breakdown of magnesite and magnetite from talc-carbonate altered ultramafic rock, mobilisation of the released Mg in the weathering runoff and re-precipitation of iron as ferric oxide and hydroxide phases. Ni solubility in recent natural waters is low^45^ and Ni released from breakdown of talc and relict serpentine is effectively sequestered in hydrous silicate and Fe- and Mn-bearing weathering product phases (Fig. 2d). This is consistent with high Ni/Mg and Si/Mg of the investigated laterite and sandstone samples and the low Ni concentration in riverine and ocean water^45,46^.

The composition of sediments used to define the average composition of the upper continental crust is controlled by rock composition in the provenance area and element solubility in surface water. Sediment concentrations of elements with low solubility in aqueous fluid, such as Ni and Cr^5^, provide boundary conditions for element transport during weathering and erosion of their host rock. We can therefore estimate both the total amount of peridotite weathering required to liberate the given total mass of Ni present in the upper continental crust as well as the peridotite weathering flux to maintain it. Nickel concentrations of the upper continental and andesitic crust^5,17^ and the average concentration of 1325 ± 888 ppm Ni in peridotite (*n* = 881)^47^ constrain the mass of peridotite required to balance the andesite model to 1.28 wt% of today’s continental crust.

Assuming that the Ni budget of the Phanerozoic continental crust is at steady-state with input from weathering of volcanic arcs and ultramafic rock and output due to sediment transport into subduction zone trenches and dissolved Ni in the continental runoff, the mass-balance provides constraints on the ultramafic rock weathering flux. The steady-state assumption can only apply for the Phanerozoic as during the Precambrian large volumes of Ni-rich komatiite were continuously being added to the crust. Furthermore, elevated atmospheric CO_2_ concentrations during the Archean^48,49^ and resulting lower pH of surface waters have likely prevented precipitation of Ni^50^ during weathering of komatiite. Hence, Archean weathering and solute transport in the surface runoff can be assumed to be distinct from the Phanerozoic.

**Fig. 10:**
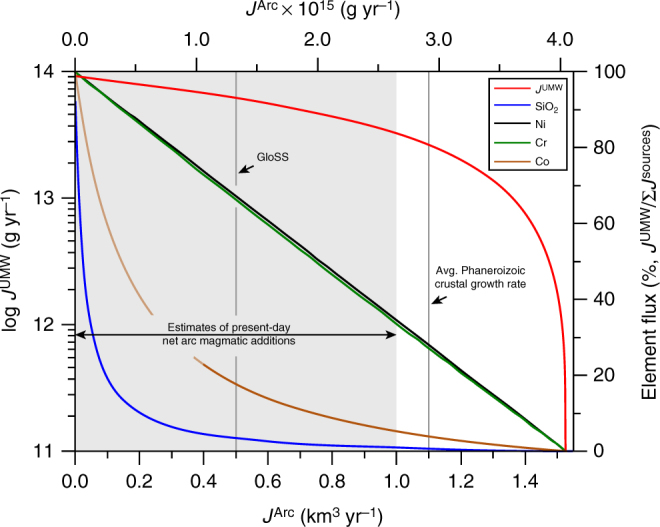
Weathering and element fluxes from ultramafic rock. The contribution of ultramafic rock weathering (*J*^UMW^) to balancing the Ni budget of the continental crust relative to the input from weathering of average andesite in volcanic arcs (*J*^Arc^) (red line, left-hand ordinate). The volcanic arc weathering flux is given as km^3^ yr^−1^ (i.e. Armstrong Units) to facilitate comparison with crustal growth rates. The secondary abscissa provides the volcanic arc weathering flux in g yr^−1^, calculated using the andesite density of 2.65 g cm^−3^. The right-hand ordinate shows the Ni, Cr, Co and SiO_2_ release from ultramafic rock weathering as percentage relative to *J*^UMW^ + *J*^Arc^. The grey shaded area represents the range of current estimates of crustal growth rates and the growth rate of 1.1 km^3^ yr^−1^ represents the average for the Phanerozoic^41, 51^. For volcanic arc weathering rates below 0.4 km^3^ yr^−1^ Co input from volcanic arc and ultramafic rock weathering is outweighed by Co removal from the continents

Figure 10 shows the ultramafic weathering flux (*J*^UMW^) required to balance Ni in the in- and output fluxes from the continental crust based on the sediment transport and riverine discharge rates and their Ni concentrations together with the Ni concentration in the average andesitic crust relative to the weathering flux from volcanic arcs (*J*^Arc^)^5,17,41,45^. The weathering flux from continental volcanic arcs is unknown but constraints can be derived from rates of arc magmatic addition and bulk sediment transport to the oceans. The current sediment removal rate from the continents is ~0.5 km^3^ yr^−1^ ^41^ while the estimated average arc accretion rate is ~1.1 km^3^ yr^−1^ ^41,51–53^. Even though these estimates are subject to uncertainty, the weathering flux from arc magmatic rocks that contributes to continental sediments must be smaller than arc accretion rates to allow for growth or maintenance of the continental crust. It is therefore probable that the accumulation rate of sediment derived from weathering of volcanic arcs is similar to the total continental sediment output or smaller. For volcanic arc weathering rates of 0.5 km^3^ yr^−1^ and 0.25 km^3^ yr^−1^, the mass-balance indicates an ultramafic weathering flux of 6.18 ± 4.46 × 10^13^ g yr^−1^ and 7.68 ± 5.40 × 10^13^ g yr^−1^, respectively (Fig. 10). In our model the ultramafic weathering flux and its contribution to the crustal Ni, Cr, Co and SiO_2_ budget scales inversely with the weathering flux from volcanic arcs. At arc weathering rates of 0.5 km^3^ yr^−1^, the ultramafic weathering flux represents only 4.4% of the total input fluxes but controls ~67% of the Ni and Cr and 18% of the Co additions to continental sediments. The contribution of Ni, Cr from ultramafic rock weathering increase to ~83% and that of Co to 35% of the total input at a volcanic arc weathering flux of 0.25 km^3^ yr^−1^ (Fig. 10). In comparison, present-day erosion denudation of the Semail ophiolite, Oman, proceeds at a rate of 0.3 mm year^−1^ ^54^ equivalent to ~4.57 × 10^12^ g yr^−1^ peridotite weathering or 7.4% of the calculated global peridotite weathering flux (at arc weathering of 0.5 km^3^ yr^−1^). The presence of several similarly large ophiolite complexes^28^ implies that our estimate of the global ultramafic weathering flux is consistent with independently constrained peridotite weathering rates in nature and corroborates the importance of ultramafic rock weathering for the Ni and Cr budget of the continental crust. The mass-balance also indicates that Cr accumulates on the continents relative to Ni or that Ni is preferentially transported to the oceans relative to Cr. This is reflected in estimates of the upper continental crust composition and Ni and Cr concentrations in banded iron formations^17,46^ (Fig. 9). In contrast to Ni and Cr, the Co concentration of the upper continental crust (17.3 ppm) is not enriched relative to average andesite (25 ppm)^5,17^. Our model indicates that the impact of ultramafic rock weathering (115 ± 81 ppm Co; *n* = 884^47^) on the crustal Co budget is small relative to the contribution of volcanic arc weathering and that Co is effectively removed from the continents at volcanic arc weathering rates below 0.4 km^3^ yr^−1^ (Fig. 10).

Even though we use the composition of average andesite^5^ in the presented mass-balance calculation, the big picture is not expected to change significantly if other rock types that are typically present in volcanic arcs are considered. The compilation of bulk rock trace-element data of volcanic arc rocks^55^ with more than 45 wt% SiO_2_ (i.e. including also mafic rocks) indicates average volcanic arc concentrations of 27.7 ± 44.2 ppm Ni (*n* = 10,194), 68.7 ± 137 ppm Cr (*n* = 9783) and 18.5 ± 12.8 ppm Co (*n* = 6310). These average concentrations are similar to those of average andesite^5^ indicating that the average andesite composition is a valid proxy for the different lithologies present in volcanic arcs. Nevertheless, the presented mass-balance represents a maximum estimate for the amount of ultramafic rock weathering contributing to continental sediments, as additional Ni is provided by weathering of continental intraplate volcanic rocks including e.g. flood basalt. The compilation of compositional data for continental flood basalt^55^ indicates average concentrations of 98 ± 125 ppm Ni and 203 ± 297 ppm Cr (*n* = 11,013). These concentrations are slightly higher than in average andesite, the bulk, and upper continental crust but significantly lower than that of peridotite. As continental weathering of mafic rocks is somewhat slower than that of ultramafic rocks^56^ and the overall contribution of continental intraplate volcanic rocks to crustal growth is presumably small (0.1 km^3^ yr^−1^ ^51^), we conclude that Ni, Cr and Co addition from weathering of intraplate volcanic rocks can only be a fraction of that derived from ultramafic rock and volcanic arc weathering.

The presence of silcrete and listvenite in altered ultramafic terranes provides direct evidence that both chemical weathering and hydrothermal carbonation favour generation of silica-enriched product phases extending to pure quartz, which is eventually added to the continental crust as sediment. High bulk rock Ni concentrations in quartz-bearing ultramafic weathering residuals and hydrothermally altered peridotite indicates that Ni can be used as a conservative tracer of fluid-driven silica release from ultramafic rock. Based on the calculated ultramafic rock weathering flux at volcanic arc weathering of 0.5 km^3^ yr^−1^, SiO_2_ liberation from altered ultramafic rock is approximately 2.78 ± 2.0 × 10^13^ g yr^−1^ that is equivalent to 3.4% of the total SiO_2_ input to the continental crust in our mass-balance calculation (Fig. 10). SiO_2_ liberation to the continental crust increases to 3.46 ± 2.43 × 10^13^ g yr^−1^ at an arc weathering rate of 0.25 km^3^ yr^−1^, which is equivalent to 8.1% of the SiO_2_ input flux. The mass-balance implies that in the absence of tectonically driven production of SiO_2_-rich melts along continental margins and/or in the presence of a larger proportion of ultramafic material during the Archaean, peridotite alteration and weathering must have been more significant contributors to silica enrichment of the evolving continental crust. The geological record shows that peridotite carbonation products including talc-carbonate and quartz-fuchsite (indicative of listvenite alteration) assemblages are common in Archaean greenstone belts (Fig. 5)^57–59^. Although their presence alone cannot constrain the timing of alteration, high Ni/Fe in Archaean banded iron formations can be related to elevated seawater Ni concentration (~400 nm) approximately 41 times higher than Phanerozoic seawater (~9 nm) resulting from chemical weathering of the proto-continental crust with a large proportion of Ni-rich peridotite^46^. Archean hydrothermal alteration and weathering has likely been facilitated by elevated atmospheric *p*_CO2_ and heat flux^18,48,49,60,61^ resulting in low pH surface waters and increased dissolution kinetics. While this particular geochemical environment would diminish the formation of Fe-hydroxide phases and low-temperature carbonates that sequester Ni from the continental runoff, it favours precipitation of crystalline and amorphous silica phases. If the production of silica-enriched weathering residuals on the continents scales with the seawater Ni concentration, chemical weathering of ultramafic and mafic rocks must have been an important driver towards the compositionally evolved felsic continental crust prior to the onset of plate tectonics.

## Methods

### The ultramafic rock weathering mass-balance

The average composition of the continental crust is based on the composition of continental sediments. Weathering of two principal source rocks contribute to the sedimentary Ni budget, low-Ni differentiated magmatic rock (andesite) and high-Ni ultramafic rock. The ultramafic source comprises komatiite flows, ultramafic intrusions and tectonically obducted oceanic lithosphere (ophiolites). The upper continental crust Ni concentration has been constrained to 47 ppm^17^, whereas average andesitic crust has 30 ppm Ni^5^ and average ultramafic rock has 1324 ± 888 ppm Ni (*n* = 881)^47^. To compensate for the low Ni concentration in andesite that contributes the bulk mass to the continental crust, Ni addition via weathering of peridotite equivalent to ~1.28 wt% of the bulk continental crust is required. The calculated mass is based on the continental crust volume of 7.18 × 10^9^ km^3^ ^5^ and andesite density of 2.65 g cm^−3^ and represents a minimum estimate as Ni is continuously removed from the continental crust by the sediment runoff and river discharge.

The contribution of ultramafic rock weathering to the composition of the continental crust can be further explored by constraining the weathering flux required to maintain its Ni budget. By restricting our flux calculation to the Phanerozoic, the mass-balance simplifies because of the relatively even occurrence of ophiolite complexes throughout the Phanerozoic^62,63^, the lack of evidence for significant Mg concentration changes of the Phanerozoic continental weathering flux^64^ and the uniform composition of post-Archaean terrigenous clastic sediments^21,60,65^. We can therefore assume that the Phanerozoic Ni budget of the continental crust is at steady state with input from weathering of accretionary volcanic arcs and ultramafic rocks and output as dissolved and particulate matter in the continental runoff^41,45^. The steady-state assumption cannot be made for the Archean due to the continuous addition of Ni-rich komatiite^66^. We can therefore define the continental mass-balance for Ni as:

1 $$\frac{{{\rm d}N_{{\rm Ni}}^{{\rm CC}}}}{{{\rm d}t}} = C_{{\rm UMW}}^{{\rm Ni}} \times J_{{\rm UMW}} + C_{{\rm Arc}}^{{\rm Ni}} \times J_{{\rm Arc}} - C_{{\rm GloSS}}^{{\rm Ni}} \times J_{{\rm GloSS}} - C_{{\rm River}}^{{\rm Ni}} \times J_{{\rm River}},$$

where $N_{{\rm Ni}}^{{\rm CC}}$ denotes the total mass of Ni in the continental crust (CC), *J* represents the fluxes for each of the inputs and outputs and *C*^Ni^ their Ni concentrations. Subscripts UMW, Arc, GloSS and River denote ultramafic rock weathering, weathering of volcanic arc-derived average andesite, global subducting sediments and the continental runoff. Based on the steady-state assumption, the ultramafic rock weathering flux can be calculated using:

2 $$J_{{\rm UMW}} = \frac{{J_{{\rm GloSS}} \times C_{{\rm GloSS}}^{{\rm Ni}} + J_{{\rm River}} \times C_{{\rm River}}^{{\rm Ni}} - J_{{\rm Arc}} \times C_{{\rm Arc}}^{{\rm Ni}}}}{{C_{{\rm UMW}}^{{\rm Ni}}}}.$$

The global flux of terrigenous sediments (*J*_GloSS_) and its Ni concentration ($C_{{\rm GloSS}}^{{\rm Ni}}$) has been estimated to 1.3 × 10^15^ g yr^−1^ and 70.5 ± 14.7 ppm^41^, $C_{{\rm Arc}}^{{\rm Ni}}$(27 ± 3 ppm) is based on the composition of the andesitic crust^5^, $C_{{\rm UMW}}^{{\rm Ni}}$ is the average Ni concentration of ultramafic rock (1324 ± 888 ppm^47^), and the average Ni concentration of the continental runoff and the global discharge are 0.801 ± 0.08 ppb and 3.74 ± 0.37 × 10^19^ g yr^−1^, respectively^45^. For our calculation we use $C_{{\rm Arc}}^{{\rm Ni}}$ = 30 ppm^5^. This concentration is consistent with the average Ni concentration of the global compilation (27.7 ± 44.2 ppm Ni; *n* = 10,194)^55^ of volcanic arc rocks with bulk rock SiO_2_ concentrations above 45 wt%, which accounts for the presence of mafic rocks in volcanic arcs. The volume to mass conversion in our model is based on the andesite density of 2.65 g cm^−3^. Calculated uncertainties for the ultramafic weathering and element fluxes are based on propagation of individual uncertainties of each flux and concentration value. We use published uncertainties for the flux and element concentrations of the global subducting sediment (GloSS)^41^, estimate a 10% uncertainty for the global riverine discharge and its concentrations^45^ and the concentrations in average andesite^5^. The uncertainty in all calculated average concentrations for ultramafic and volcanic arc rocks is the 1σ standard deviation and represents the overall spread of the data population^47,55^.

### Electron probe micro analysis

Quantitative elemental maps and mineral compositions were acquired on a JEOL 8530F electron probe micro analyser (EPMA) housed at the Centre for Microscopy, Characterisation and Analysis (CMCA), The University of Western Australia (UWA). The EPMA is equipped with five tunable wavelength dispersive spectrometers. Operating conditions were 40 degrees takeoff angle, and a beam energy of 15 keV. The beam current was 20 nA for calibration and map acquisition. The beam diameter was 2 µm. Dwell time was 40 msec per pixel with a pixel dimension of 2 × 2 µm. Elements were acquired using analysing crystals LiFH for Ti Kα1, Cr Kα1, Mn Kα1, LiF for Fe Kα1, Ni Kα1, PETJ for Ca Kα1, K Kα1, and TAP for Mg Kα1, Si Kα1, Al Kα1, Na Kα1. The standards were an assortment of synthetic and natural minerals and metals. The counting time was 20 s on peak for all elements, and Mean Atomic Number background corrects were used throughout^67^. The intensity data was corrected for Time Dependent Intensity (TDI) loss (or gain) using a self–calibrated correction for Si Kα1, Na Kα1, Ti Kα1, K Kα1, Fe Kα1. Interference corrections were applied to Fe for interference by Mn, and to Mn for interference by Cr^68^. Results are the average of three points and detection limits ranged from 0.006 wt% for Si Kα1 to 0.008 wt% for Al Kα1 to 0.009 wt% for Na Kα1 to 0.012 wt% for Ti Kα1 to 0.028 wt% for Ni Kα1. Analytical sensitivity (at the 99% confidence level) ranged from 0.279% relative for Si Kα1 to 0.981% relative for Al Kα1 to 10.056% relative for Cr Kα1 to 52.104% relative for Na Kα1 to 392.577% relative for Ti Kα1. Oxygen was calculated by cation stoichiometry and included in the matrix correction. The elemental maps were processed using Probe Software’s CalcImage application and Matlab. The matrix correction method was ZAF and the mass absorption coefficients data set was LINEMU Henke (LBL, 1985) < 10 KeV/CITZMU > 1 0KeV. The ZAF algorithm utilised was Armstrong/Love Scott^69^.

Qualitative elemental maps were acquired using a Cameca SX100 electron microprobe (Institute of Geosciences, University of Oslo). Analyses were carried out using an acceleration voltage of 15 kV, a beam current of 10 nA, and a counting time of 10 s for major and minor elements. Na and K were analysed first. Standardisation was done with synthetic oxides and natural minerals. Matrix corrections follow described procedures^70^, which are implemented in the CAMECA PAP‐program.

### Mg isotope analysis

The magnesium isotopic composition of the whole rock samples was determined after chromatographic separation following the protocol described in Mavromatis et al.^71^. Magnesium separation (~20 µg) of bulk digests took place in 10 ml Bio-Rad Poly-prep columns and >99% of the Mg loaded into the columns was recovered at the end of the procedure. The cation/Mg ratio of all samples prior to isotope analysis was <0.001 as determined by ICP–MS analysis. Magnesium isotopic compositions were measured using a Thermo-Finnigan ‘Neptune’ Multi Collector ICP–MS at Géosciences Environnement Toulouse (GET), France. All solutions were prepared in 0.32 m HNO_3_ and introduced into the argon plasma using a standard spray chamber. Solution concentrations were typically ~600 ppb and gave intensities of ~12 V, with total procedural blanks generally having a negligible contribution of <3 mV. Sample-standard bracketing was used to correct instrumental mass fractionation effects and all data are presented as *δ*^*x*^Mg with respect to DSM3 international reference material (*δ*^*x*^Mg = ((^*x*^Mg/^24^Mg)_sample_/(^*x*^Mg/^24^Mg)_DSM3_−1) × 1000), where *x* refers to the Mg mass of interest. All sample analyses were run in triplicate with the mean value presented in Supplementary Table 1. The reproducibility of *δ*^26^Mg analyses was assessed by replicate analyses of Mg reference standards reported in Supplementary Table 1 and was typically better than 0.08‰. Moreover, dolomite standard JDo-1 and Mg standard CAM-1 were identically processed resulting in compositions similar to those reported elsewhere^72–74^.

### Bulk rock chemical analysis

Whole-rock geochemical analyses including CO_2_ and FeO were performed by Actlabs Laboratories Ltd., using the lithium metaborate/tetraborate fusion ICP Whole Rock and the trace element ICP/MS packages.

Samples are mixed with a flux of lithium metaborate and lithium tetraborate and fused in an induction furnace. The melt is immediately poured into a solution of 5% nitric acid containing an internal standard, and mixed continuously until completely dissolved (~30 min). The samples are run for major oxides and selected trace elements on a combination simultaneous/sequential Thermo Jarrell–Ash ENVIRO II ICP or a Varian Vista 735 ICP. Calibration is performed using seven prepared USGS and CANMET certified reference materials. One of the seven standards is used during the analysis for every group of ten samples. FeO is determined through titration, using a cold acid digestion of ammonium metavanadate, and hydrofluoric acid in an open system. Ferrous ammonium sulphate is added after digestion and potassium dichromate is the titrating agent. Weight fractions of dry CO_2_ sample gas are measured by infrared absorption after decomposing 0.2 g of sample material in a resistance furnace in a pure nitrogen environment at 1000 °C, using an ELTRA CW–800 (www.actlabs.com).

### Data availability

The data used to support the findings of this study are available from the corresponding author upon request.

## Electronic supplementary material

Supplementary Information

Description of Additional Supplementary Files

Supplementary Data 1

Supplementary Data 2
