# Supplementary material for: Peridotite weathering is the missing ingredient of Earth’s continental crust composition
Source: Nat Commun. 2018 Feb 12;9:634. doi: 10.1038/s41467-018-03039-9 (PMC5809581; doi:10.1038/s41467-018-03039-9)
Supplement: Supplementary file 1 — Supplementary Information [file 41467_2018_3039_MOESM1_ESM.pdf]

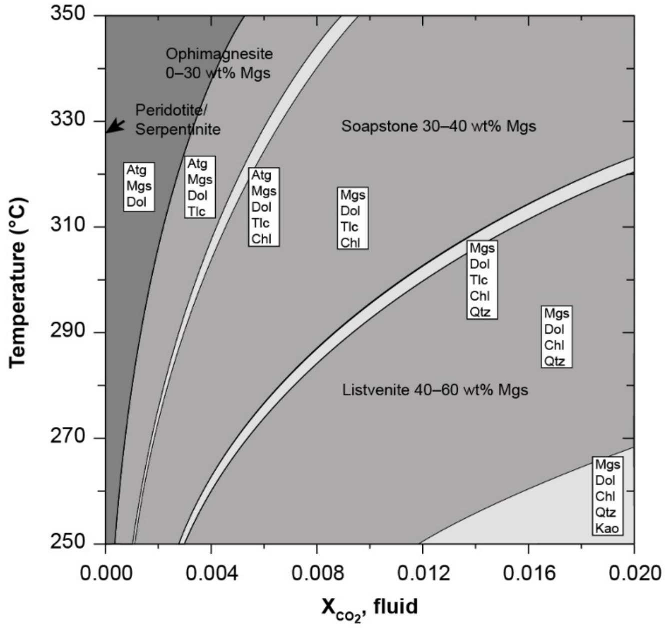

Supplementary Figure 1 Phase relationships in ultramafic CO_2 alteration systems. Isobaric T-XCO_2 phase diagram for hydrothermal peridotite carbonation calculated for the system CaO–FeO–MgO–Al_2O_3–SiO_2–H_2O–CO_2 using the software package Perple_X [1] at 3 kbar. The SiO_2 content of silicate phases and carbonate abundance increase with XCO_2 content of the alteration fluid. Surface weathering will preferentially remove magnesite from the alteration product assemblage and weathering of listvenite will result in the formation of quartz–bearing sediment. The phase diagram is calculated using the composition of pristine serpentinite from Linnajavri, Norway (Lin_31) [2].

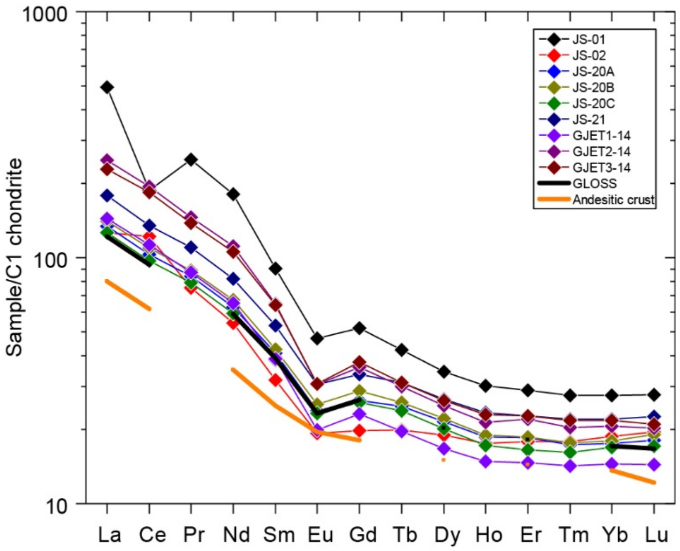

Supplementary Figure 2 Chemical characteristics of peridotite derived sandstone. C1 chondrite–normalised REE concentrations [3] of weathered peridotite–bearing sandstone samples from Solund and Feragen, Global Subducting Sediments (GloSS) [4], and the average andesitic crust [5].

Supplementary Table 1. Mg isotope data of drill core samples from the Six Mile Well–Goliath Complex, Western Australia.

Sample	Description	Rock type	Depth (m)	δ^{25}Mg (‰)	2σ	δ^{26}Mg (‰)	2σ
G1	Bulk sample	Serp.	119.2	-0.07	0.05	-0.14	0.07
G2	Bulk sample	Serp.	109.6	-0.10	0.04	-0.24	0.07
G3	Bulk sample	Ophimag.	99.4	-0.14	0.03	-0.30	0.05
G4a	Bulk sample	Ophimag.	90.4	-0.04	0.02	-0.10	0.03
G4b	Vein magnesite	Ophimag.	90.4	-0.06	0.04	-0.12	0.04
G5	Bulk sample	Ophimag.	87.2	-0.16	0.02	-0.31	0.04
G6	Bulk sample	Ophimag.	79.5	-0.13	0.01	-0.28	0.01
G7	Bulk sample	Felsic dike	66.5	-0.30	0.02	-0.59	0.05
G8a	Bulk sample	Tlc-carb	54.3	-0.26	0.03	-0.51	0.07
G8b	Bulk sample	Tlc-carb	54.3	-0.31	0.04	-0.60	0.05
G9a	Dark patch	Mafic dike	44.5	-0.64	0.04	-1.21	0.07
G9b	Bulk sample	Mafic dike	44.5	-0.13	0.00	-0.28	0.05
G10	Bulk sample	Tlc-carb	33	-0.34	0.03	-0.67	0.06
G11	Bulk sample	Tlc-carb	26.3	-0.20	0.02	-0.42	0.07
G12a	Vein magnesite	Tlc-carb weath.	26.2	-0.35	0.05	-0.68	0.05
G12b	Bulk sample	Tlc-carb weath.	26.2	-0.01	0.02	-0.04	0.01
G13	Bulk sample	Laterite	17.8	0.24	0.05	0.46	0.05
G14	Bulk sample	Laterite	5.2	0.28	0.04	0.54	0.03
G15a	Bulk sample	Laterite	0.3	0.21	0.01	0.42	0.02
G15b	Bulk sample	Laterite	0.3	0.20	0.02	0.37	0.01
G2	Replicate			-0.12	0.04	-0.24	0.05
G8	Replicate			-0.30	0.03	-0.61	0.06
G13	Replicate			0.23	0.04	0.46	0.03
CAM1	Reference material			-1.32	0.05	-2.63	0.06
OUMg	Reference material			-1.42	0.04	-2.80	0.05
BE-N	Reference material			-0.13	0.07	-0.26	0.07
JDo1	Reference material			-1.21	0.05	-2.42	0.08
IAPSO	Reference material			-0.41	0.03	-0.85	0.05

*Serp. = serpentinite; Ophimag. = ophimagnesite; Tlc-carb = talc-carbonate altered serpentinite; Tlc-carb weath. = weathered talc-carbonate rock.

Supplementary Table 2. Bulk rock major and trace-element composition of sandstone samples from Norwegian peridotite-bearing sedimentary basins.

Sample<br>Location	GJET1-14<br>Feragen	GJET2-14<br>Feragen	GJET3-14<br>Feragen	JS-01<br>Solund	JS-02<br>Solund	JS-20A<br>Solund	JS-20B<br>Solund	JS-20C<br>Solund	JS-21<br>Solund	Detection Limit
SiO_2 wt%	67.92	64.66	66.71	59.55	64.48	62.66	63.05	62.67	55.38	0.01
Al_2O_3	13.33	15.53	14.69	12.74	13.22	12.3	12.56	12.01	11.75	0.01
Fe_2O_3_total	6.5	7.01	6.41	7.92	7.17	6.89	7.09	6.69	6.93	0.01
MnO	0.08	0.081	0.07	0.157	0.132	0.115	0.115	0.114	0.154	0.001
MgO	3.47	3.23	2.99	4.82	5.14	5.08	5.08	5.05	4.23	0.01
CaO	0.19	0.33	0.42	4.7	2.33	4.35	3.86	3.59	9.51	0.01
Na_2O	1.27	1.34	1.48	2.25	2.33	2.15	2.19	2.12	2.02	0.01
K_2O	2.9	3.7	3.52	1.75	1.96	1.67	1.63	1.7	1.89	0.01
TiO_2	0.685	1.03	0.972	1.135	0.9	1.038	1.083	0.963	1.129	0.001
P_2O_5	0.09	0.14	0.15	0.19	0.14	0.14	0.15	0.13	0.22	0.01
L.O.I	3.06	3.3	3.03	3.47	2.96	4.53	4.02	4.14	7.56	
Total	99.5	100.3	100.5	98.67	100.8	100.9	100.8	99.18	100.8	0.01
Sc ppm	12	15	14	21	17	18	18	17	19	1
Be	2	3	3	2	2	2	2	2	2	1
V	93	115	104	138	108	132	136	128	155	5
Cr	150	120	100	280	360	190	210	210	180	20
Co	19	19	16	23	25	23	24	24	22	1
Ni	140	90	70	140	210	160	160	180	140	20
Cu	20	20	20	40	30	30	30	30	50	10
Zn	80	90	80	100	90	80	90	80	90	30
Ga	17	20	19	16	14	16	16	16	16	1
Ge	2.1	2.2	2.2	2.2	1.7	1.4	1.6	1.5	1.5	0.5
As	< 5	6	7	< 5	< 5	< 5	< 5	< 5	< 5	5
Rb	106	136	131	55	60	55	54	56	64	1
Sr	34	48	52	303	252	155	166	138	195	2
Y	21.8	31.8	33.2	50.5	28.8	31.2	32.6	29.2	39.4	0.5
Zr	196	330	316	310	280	211	224	215	326	1
Nb	8.9	15.6	13.4	26.2	24	14.8	15.6	14.6	16.3	0.2
Mo	< 2	< 2	< 2	< 2	< 2	< 2	< 2	< 2	< 2	2
Ag	0.6	1.3	1.3	0.9	0.9	0.6	0.6	0.6	1.1	0.5
In	< 0.1	< 0.1	< 0.1	< 0.1	< 0.1	< 0.1	< 0.1	< 0.1	< 0.1	0.1
Sn	2	3	3	2	2	2	2	2	2	1
Sb	0.9	1.3	1.3	0.2	0.3	0.3	0.5	0.3	0.5	0.2
Cs	3.4	4.8	4.7	0.8	1	0.9	1	1	0.9	0.1
Ba	345	447	417	493	564	449	410	413	434	3
La	34.2	59	54.3	117	29.9	31.8	33.4	29.9	42.4	0.05
Ce	69	119	113	115	74.9	63.2	66.9	59.5	82.6	0.05
Pr	8.07	13.5	12.8	23.2	6.99	7.78	8.23	7.33	10.2	0.01
Nd	29.9	50.8	48.2	82.6	24.8	28.8	30.7	27.1	37.6	0.05
Sm	5.73	9.59	9.51	13.4	4.7	6.04	6.29	5.67	7.85	0.01
Eu	1.12	1.72	1.73	2.64	1.09	1.34	1.43	1.31	1.72	0.005
Gd	4.61	7.13	7.5	10.3	3.94	5.23	5.71	5.12	6.68	0.01
Tb	0.71	1.08	1.12	1.52	0.72	0.9	0.93	0.86	1.12	0.01
Dy	4.12	6.17	6.45	8.47	4.68	5.3	5.45	4.95	6.52	0.01
Ho	0.81	1.17	1.26	1.65	0.96	1.02	1.04	0.94	1.28	0.01
Er	2.35	3.53	3.63	4.62	2.87	2.96	2.99	2.65	3.63	0.01
Tm	0.352	0.505	0.539	0.679	0.442	0.43	0.437	0.4	0.545	0.005
Yb	2.34	3.32	3.51	4.44	3.03	2.83	2.9	2.73	3.56	0.01
Lu	0.355	0.499	0.516	0.68	0.482	0.445	0.469	0.423	0.556	0.002
Hf	4.4	7.6	7.2	6.9	6.3	4.7	5.1	5	7.4	0.1
Ta	1	1.45	1.33	1.8	1.65	1.02	1.04	0.97	1	0.01
W	2.1	2.3	2.2	< 0.5	< 0.5	0.6	< 0.5	< 0.5	< 0.5	0.5
Tl	0.49	0.68	0.62	0.22	0.23	0.18	0.2	0.2	0.25	0.05
Pb	14	19	17	21	16	9	9	8	13	5
Bi	0.2	0.2	0.2	0.1	< 0.1	< 0.1	0.7	< 0.1	< 0.1	0.1
Th	10.8	17.4	16.2	13	12	7.3	7.69	6.83	9.4	0.05
U	2.12	2.59	3	2.72	2.93	1.74	2.27	1.81	2.83	0.01

Supplementary Table 3. Average composition of major minerals in drill core of the Six Mile Well–Goliath Complex, Western Australia.

	serpentine		talc		magnesite		chlorite	
	n = 47		n = 25		n = 11		n = 12	
	wt%	1σ	wt%	1σ	wt%	1σ	wt%	1σ
SiO_2	43.31	1.25	63.51	1.13	0.03	0.11	33.29	1.17
Al_2O_3	0.31	0.40	0.06	0.06	0.00	0.01	12.01	4.36
TiO_2	0.01	0.01	0.05	0.07	0.03	0.05	0.02	0.02
FeO	2.02	1.98	1.97	0.43	7.28	1.11	7.69	5.62
MnO	0.02	0.02	0.00	0.01	0.10	0.04	0.26	0.42
MgO	40.64	2.11	29.24	0.56	38.42	0.64	28.63	4.80
CaO	0.01	0.02	0.01	0.03	0.13	0.02	0.07	0.09
Na_2O	0.16	0.49	0.05	0.04	0.00	0.00	0.17	0.22
K_2O	0.02	0.12	0.01	0.01	0.00	0.00	0.08	0.11
Cr_2O_3	0.07	0.07	0.02	0.02	0.00	0.00	0.69	0.79
NiO	0.27	0.17	0.25	0.04	0.00	0.00	3.21	5.01
F	n.d.	n.c.	0.05	0.05	0.00	0.00	n.d.	n.c.
Cl	n.d.	n.c.	0.02	0.02	0.00	0.00	n.d.	n.c.
H_2O*	n.c.	n.c.	4.71	0.02	n.c.	n.c.	n.c.	n.c.
Total	86.83	1.12	99.97	0.82	46.01	0.54	86.12	1.30
O	7.00		12.00		1.00		28.00	
Si	2.02	0.04	4.04	0.04	0.00	0.00	6.58	0.27
Al	0.01	0.01	0.00	0.00	0.00	0.00	2.78	0.96
Ti	0.00	0.00	0.00	0.00	0.00	0.00	0.00	0.00
Fe^{2+}	0.08	0.08	0.11	0.02	0.10	0.01	1.26	1.29
Fe^{3+}	n.c.	n.c.	n.c.	n.c.	n.c.	n.c.	0.11	0.11
Mn	0.00	0.00	0.00	0.00	0.00	0.00	0.05	0.07
Mg	2.83	0.14	2.78	0.06	0.90	0.01	8.39	1.11
Ca	0.00	0.00	0.00	0.00	0.00	0.00	0.02	0.02
Na	0.01	0.02	0.01	0.01	0.00	0.00	0.14	0.17
K	0.00	0.00	0.00	0.00	0.00	0.00	0.04	0.05
Cr	0.00	0.00	0.00	0.00	0.00	0.00	0.11	0.12
Ni	0.01	0.01	0.01	0.00	0.00	0.00	0.54	0.86
F	n.c.	n.c.	0.01	0.01	0.00	0.00	n.c.	n.c.
Cl	n.c.	n.c.	0.00	0.00	0.00	0.00	n.c.	n.c.
H	n.c.	n.c.	2.00	0.02	n.c.	n.c.	n.c.	n.c.
Total	4.96	0.05	8.96	0.04	1.00	0.00	20.02	0.42
Mg#	0.97	0.03	0.96	0.01	0.90	0.01	0.88	0.09
Ni (ppm)	2124	1317	1968	309	n.c.	n.c.	25234	39405

* H assigned stoichiometrically to excess oxygen.

n.c. = not calculated

n.d. = not determined

Supplementary References:

1. Connolly, J. A. D. Multivariable phase diagrams; an algorithm based on generalized thermodynamics. *American Journal of Science* **290**, 666-718 (1990).
2. Beinlich, A., Plümper, O., Hövelmann, J., Austrheim, H. & Jamtveit, B. Massive serpentinite carbonation at Linnajavri, N-Norway. *Terra Nova* **24**, 446-455 (2012).
3. McDonough, W. F. & Sun, S. S. The composition of the Earth. *Chemical Geology* **120**, 223-253 (1995).
4. Plank, T. & Langmuir, C. H. The chemical composition of subducting sediment and its consequences for the crust and mantle. *Chemical Geology* **145**, 325-394 (1998).
5. Taylor, S. R. & McLennan, S. M. The geochemical evolution of the continental crust. *Rev Geophys* **33**, 241-265 (1995).
