# Supplementary material for: Peridotite weathering is the missing ingredient of Earth’s continental crust composition
Source: Nat Commun. 2018 Feb 12;9:634. doi: 10.1038/s41467-018-03039-9 (PMC5809581; doi:10.1038/s41467-018-03039-9)
Supplement: Supplementary file 2 — Description of Additional Supplementary Files [file 41467_2018_3039_MOESM2_ESM.pdf]

Description of Additional Supplementary Files

File Name: Supplementary Data 1
Description: Supplementary Data 1 contains the bulk rock composition, calculated carbonate content, and SiO_2, Cr and Ni concentrations on a carbonate-free basis. The data is shown in Fig. 5.

File Name: Supplementary Data 2
Description: Supplementary Data 2 contains the bulk rock major and trace-element composition, depth and description of drill core samples from the Six Mile Well–Goliath Complex, Western Australia.
